# Disability inclusion in rural primary education in Malawi: A case study of knowledge, attitudes and practices

**DOI:** 10.4102/ajod.v15i0.2016

**Published:** 2026-10-14

**Authors:** Priscilla G. Banda, Ben de Souza, Richard Vergunst

**Affiliations:** 1 Division of Disability Studies, Department of Health and Rehabilitation Services, Faculty of Health Sciences, University of Cape Town, Cape Town, South Africa; 2 Department of Secondary and Post-School Education, Faculty of Education, Rhodes University, Makhanda, South Africa; 3 Department of Psychology, Faculty of Humanities, Independent Institute of Education, Emeris Newlands, Cape Town, South Africa

**Keywords:** inclusive education, rural schooling, Malawi, disability, stakeholder knowledge, teacher attitudes, collaboration, special educational needs

## Abstract

**Background:**

Malawi’s education policy endorses inclusive education. However, there is limited empirical evidence on how stakeholder knowledge, attitudes and structural conditions interact to shape inclusive education in rural school settings.

**Objectives:**

This study explored how stakeholder knowledge, attitudes and contextual factors influence the implementation of inclusive education for learners with disabilities in a rural government primary school in Kasungu District, Malawi.

**Method:**

A qualitative case study design was employed. Data were generated through interviews and a focus group discussion involving teachers, school leaders, parents and community stakeholders. Data were analysed thematically, informed by the social model of disability and the Knowledge–Attitudes–Practices framework.

**Results:**

Inclusive education was constrained by limited policy awareness, inadequate teacher preparation, shortages of specialist support, inaccessible infrastructure and disability-related stigma. Although stakeholders generally supported inclusion, their confidence in supporting learners with complex disabilities was shaped by structural constraints. Teacher commitment, supportive leadership and community engagement facilitated inclusive practices but were insufficient to overcome systemic barriers.

**Conclusion:**

The findings demonstrate that inclusive education in rural Malawi is shaped by the interaction between stakeholder agency and structural conditions. Strengthening inclusive education therefore requires coordinated investment in teacher development, institutional support, accessible learning environments and intersectoral collaboration.

**Contribution:**

This study provides context-specific evidence on how knowledge, attitudes and systemic factors jointly influence inclusive education in rural Malawi, extending understanding of the complex relationship between stakeholder agency and structural constraints in low-resource settings.

## Introduction

Inclusive education is now a prominent feature in policy, practice and research. Countries have formulated policies to guide inclusive education practice. Equally, researchers have explored ways to improve inclusive education policy and strengthen implementation. In Malawi, research on inclusive education has attracted consideration attention, but most of it focuses on stakeholder awareness or policy analysis (e.g. Banda 2024; Lingolwe & Chirwa 2021). These studies and many others found that most stakeholders are not aware of their roles in inclusive education. At the policy level, the studies reveal that the framing of inclusivity in the policy discourse is somehow cacophonous. However, these findings offer limited insights into how knowledge and attitudes interact with structural constraints to influence inclusive practices in rural schools.

This study examines how inclusive education is enacted in a rural primary school in Kasungu District, Malawi. The study explores how stakeholders’ knowledge and attitudes towards disability intersect with material conditions, institutional arrangements, and social norms to support or hinder inclusive education in practice. The study uses the social model of disability and the Knowledge–Attitudes–Practices (KAP) model as theoretical and analytical frameworks. The social value of this study lies in amplifying the voices of those directly involved in implementing inclusive education in under-resourced rural school contexts. Scientifically, the study advances understanding of how individual agency and structural factors jointly determine inclusive education outcomes.

### Aim and questions

The study aims to examine how stakeholders’ knowledge, attitudes and contextual factors affect the implementation of inclusive education for learners with disabilities in a rural government primary school in Kasungu District, Malawi. Specifically, the study responds to three questions:

- *How does stakeholder knowledge shape their inclusive education practices?*
- *How do stakeholder attitudes influence inclusive education implementation?*
- *Based on these, how can inclusive education be improved in rural schools?*

## Literature review

Inclusive education is recognised globally as a rights-based, systemic reform agenda, grounded in frameworks such as the Salamanca Statement, the United Nations Convention on the Rights of Persons with Disabilities (CRPD) and Sustainable Development Goals (SDGs) 4 (Quality Education). It aims to ensure equitable access, participation, and achievement for all learners, including those with disabilities, through systemic change rather than individual accommodations (Ainscow 2020; United Nations Educational, Scientific and Cultural Organization [UNESCO] 2020). This requires transformation in curriculum, teaching methods, school culture and policies, not simply placing learners with disabilities in mainstream classrooms. In many high-income countries, inclusive education advances through comprehensive policies, continuous teacher development and multi-agency support. For instance, Finland integrates inclusive education into pre-service teacher training to emphasise differentiated instruction, co-teaching and special needs pedagogy (Ainscow 2020; Jahnukainen 2011). Countries like Canada and the United States promote inclusive education via continuous professional development and laws such as the Individuals with Disabilities Education Act (IDEA) (Tomlinson 2021).

Nonetheless, research shows persistent challenges. Studies in the United States and the United Kingdom (UK) report that teachers feel underprepared to support learners with complex disabilities despite strong policy stipulations for their inclusion in mainstream education (Alharbi & Iqtadar 2024; Saloviita & Consegnati 2019). This situation exposes a gap between policy goals and classroom realities.

In low-income and Southern African contexts, the policy–practice gap is even wider. Systems face limited resources, crowded classrooms and underfunded teacher training (Genovesi et al. 2022; Walton 2018). Research across Southern Africa highlights inadequate teacher training, lack of assistive devices and poor institutional coordination as major hurdles (Chitiyo & Dzenga 2021). In South Africa, despite progressive policies such as Education White Paper 6, implementation is hampered by lingering inequalities, unclear operational tasks, and limited teacher support (Engelbrecht et al. 2015; Walton & Engelbrecht 2022). Teachers see inclusivity narrowly as learner placement and not as pedagogical transformation (Engelbrecht et al. 2015). Similar issues prevail in Botswana, where teachers struggle with curriculum adaptation, classroom management and resource shortages despite policies supporting inclusion (Kuyini et al. 2024; Otukile-Mongwaketse, Mangope & Kuyini 2016). In Eswatini, Mpofu and Mkhatshwa (2024) observe that, while policies endorse inclusion, rural schools face infrastructure shortages and limited access to assistive technologies.

Teacher unpreparedness in inclusive education is a major concern in Africa. Studies from Uganda (Nantongo 2019) and Tanzania (Tungaraza 2014) show that theoretical knowledge from training is not enough without ongoing practical support. Zimbabwe and Kenya report issues of overcrowded classrooms and limited pedagogical resources, which limit teachers’ ability to effectively include learners with complex educational needs (Ohba & Malenya 2022). To address these challenges, scholars (including Walton 2018; Abdulrahman et al. 2021; Walton 2018) advocate decolonial and context-sensitive approaches to inclusion that prioritise local knowledge, community participation and culturally relevant pedagogies.

Inclusive education broadly involves addressing learner diversity by improving participation and reducing exclusion in education systems (UNESCO 2020). Based on the social model of disability, scholars highlight that exclusion mainly stems from social, institutional and attitudinal barriers, not individual impairments (Slee 2018; Walton 2018). Therefore, inclusive education requires adaptable and responsive learning environments. Common practices include differentiated instruction, curriculum flexibility, collaborative teaching, peer support and assistive technologies (Hunt 2021; Kuyini et al. 2024). Differentiated instruction, in particular, allows teachers to modify content, processes and assessments to diverse learner needs (Tomlinson 2021). However, effective implementation of these practices depends on supportive school cultures and strong relationships among teachers, families and communities (Florian 2014; Walton & Engelbrecht 2022). Eventually, inclusive education is both pedagogical and relational knowledge practice (Walton 2025).

Malawi associates with global frameworks such as the CRPD and SDG 4 and has introduced national strategies, including the National Strategy on Inclusive Education, aimed at improving teacher training, infrastructure and community involvement (MacJessie-Mbewe et al. 2023). Hitherto, empirical research shows a substantial gap between policy and practice (De Souza 2024; Lingolwe & Chirwa 2021). Studies in Malawian primary schools reveal that many teachers have only superficial exposure to inclusion during pre-service training and limited ongoing professional development, especially in rural areas (Phiri 2021). Infrastructure deficits include a lack of ramps, accessible toilets and learning materials (Banks et al. 2022).

Many other factors have also contributed to challenges in realising inclusive education in Malawi. One such factor is a lack of assistive devices for learners with sensory disabilities. Assistive technologies are scarce and disproportionately distributed in rural areas, thereby affecting learners with sensory and physical disabilities (MacJessie-Mbewe et al. 2023; eds. Werning et al. 2016). Cultural beliefs about disability, sometimes seen as misfortune or incapacity, also contribute to low expectations among communities and educators (Chilemba 2013; Lingolwe & Chirwa 2021). Amid all these challenges, weak coordination between teachers, school leaders, district officials and communities impedes sustainable support and accountability (De Souza 2022; MacJessie-Mbewe et al. 2023). While some non-governmental organisation (NGO)-led initiatives show promise with low-cost and locally based strategies such as peer tutoring and tactile aids, these efforts are largely uncoordinated, under-researched and unavailable in rural areas (Banks et al. 2022).

## Theoretical framework

This study is grounded in the social model of disability. This perspective offers a lucid view of how disability-focused inclusive education is shaped by structural conditions and stakeholder agency in a rural Malawian school. The social model sees disability not as an inherent individual deficit but as a result of social, environmental and institutional barriers that limit participation (Shakespeare 2010). Exclusion, from this perspective, stems from inaccessible infrastructure, inflexible curriculum, inadequate teacher training and negative or stigmatising attitudes and not learners’ impairments. This framework, widely used in disability and inclusive education research, challenges medicalised, deficit-focused approaches and emphasises systemic change as essential to inclusion (Slee 2018; Walton 2018). It is especially relevant in low-income, rural areas such as Malawi, where poverty, under-resourced schools and limited institutional capacity can normalise exclusion. Applying this model highlights how school environments, policies and practices actively create disabling conditions while also revealing opportunities for dismantling barriers through inclusive teaching, collaboration and community involvement. Crucially, the social model helps identify not only constraints but also practices and relationships that promote inclusion despite material limitations.

While the social model of disability explains why exclusion persists, it offers less guidance on how individual and collective actions shape everyday practices. To fill this gap, the study employs the KAP model as an analytical tool (Garner et al. 2020). The KAP model suggests that what people know influences their thoughts, which in turn shape their attitudes and practices. In education research, it has been used to explore how stakeholders’ beliefs and understandings translate into concrete behaviours in institutions. Like the social model of disability, the KAP model does not place responsibility solely on individuals but situates stakeholder practices in enabling or disabling environments.

When combined with the social model, the KAP model allows analysis of how teachers’, parents’ and school leaders’ knowledge about inclusive education and disability shapes their attitudes, expectations and responses to learners with disabilities. It also facilitates examining how these practices are influenced by structural factors such as limited resources, lack of training and policy ambiguity. Through the social and KAP models, the study conceives inclusive education as a dynamic interaction between structural conditions and stakeholder agency. This merged framework offers a basis for analysing how inclusion is negotiated, constrained and enacted in a rural Malawian primary school and advances disability discourse by focusing on lived processes that sustain or disrupt inclusion in practice, beyond policy and attitudinal surveys.

## Research methods and design

### Study design

This study used a qualitative single case study (see eds. Cohen, Manion & Morrison 2018; Stanley 2023) to explore how inclusive education for learners with disabilities was applied in a rural primary school in Malawi. A qualitative approach suited the focus on understanding stakeholders’ experiences, meanings and practices in a specific socio-educational context.

Guided by an interpretive paradigm (eds. Cohen et al. 2018), the study aimed to investigate how inclusion was negotiated in daily rural school life. The case study enables a detailed, context-specific analysis of the interaction between stakeholder knowledge, attitudes and practices, as well as structural factors influencing inclusion.

### Setting

The research was conducted at a government-operated primary school in a rural area of Kasungu District, Central Malawi. The school had roughly 1400 learners and faced significant challenges to inclusion, including high pupil–teacher ratios (about 1:120), limited specialist support, poor infrastructure and minimal access to assistive technologies. The nearest specialised school for visually impaired learners, which is supposed to act as a resource centre for the mainstream school, was about 82 km away. This distance highlights the scarcity of educational support options for learners with disabilities. The community is mainly Chewa-speaking, with Chichewa as the local and national language. Livelihoods rely mostly on subsistence farming and informal trade, and socio-economic hardship is common. This context was critical for examining inclusive education amid structural poverty and limited institutional capacity.

### Study population and sampling

Participants included key educational and community stakeholders who were directly or indirectly involved in inclusive education at the school. These comprised classroom teachers, school leadership (head and deputy head), school management committee members, parents and caregivers of learners with disabilities, community leaders and local support representatives. Both male and female participants were involved in the study. The youngest participant was 35 years old, while the oldest was 56 years old.

Eligibility required involvement in education for learners with disabilities and voluntary participation. Purposive sampling identified information-rich individuals (Ahmad & Wilkins 2025), followed by maximum variation sampling to ensure diverse stakeholder roles. With help from community gatekeepers, such as the head teacher, school counsellors and village chiefs, 30 potential participants were identified and invited via information letters and consent forms in Chichewa. After a community briefing, 15 people expressed willingness to participate. From these, 10 people were selected based on stakeholder role diversity, availability and informed consent. Of the 10 participants involved in the study, five were male and five were female. The final sample ensured representation from school, family and community.

### Data generation

Data were generated through 10 semi-structured, in-depth interviews and one focus group discussion (FGD). These methods encouraged participants to reflect on personal experiences, discuss shared challenges and explore inclusive practices. Before formal data generation, a pilot study with one teacher and one parent outside the main sample tested the clarity, cultural appropriateness, and order of the interview guide (Malmqvist et al. 2019). Minor adjustments were made based on feedback. Interviews were conducted in natural settings (e.g., at school for teachers, at home for parents), lasted 30–45 min and used Chichewa to ensure participant comfort and clear communication. Prompts focused on participants’ knowledge of inclusive education, attitudes towards disability, perceived roles and everyday practices. All interviews were audio-recorded with consent and complemented by field notes capturing contextual details. The FGD included eight participants (four males and four females) from different stakeholder groups and was conducted in a neutral community space. It lasted about an hour and was facilitated in Chichewa with inclusive techniques to promote equal participation. The FGD aimed to triangulate interview data and stimulate collective reflection on inclusion and exclusion experiences.

### Data analysis

Recordings were transcribed verbatim and translated into English, with translations checked using a bilingual dictionary and verified by a Chichewa–English language expert to preserve semantic accuracy. Data were analysed using Braun and Clarke’s (2006, 2023) six-step thematic analysis: familiarisation with the data, generating initial codes, searching for themes, reviewing themes, defining and naming themes and producing the final report.

An inductive coding approach allowed patterns to emerge from the data (Azungah 2018). Initial codes were generated through repeated readings of the transcripts and field notes, before being compared, refined and grouped into broader categories. These categories were then reviewed iteratively across the dataset and consolidated into themes that captured recurring patterns in stakeholders’ experiences and perspectives. The resulting themes were subsequently interpreted through the social model of disability and the KAP framework to examine how stakeholder knowledge and attitudes shaped inclusive education practices in structurally constrained contexts.

To enhance trustworthiness, the study employed methodological triangulation by comparing evidence across interviews, the FGD and field notes. Verbatim quotations supported interpretations, while extended engagement in the field enabled a richer understanding of the study context. Reflexive field notes were maintained throughout data generation and analysis to document emerging interpretations and critically examine our assumptions and potential influence on the analytical process. As researchers, we remained mindful of our role in interpreting participants’ accounts and sought to ensure that themes were grounded in the data.

### Ethical considerations

Ethical approval was obtained from the University of Cape Town’s Human Research Ethics Committee (HREC REF: 128/2022) and relevant authorities in Malawi, including the Ministry of Education and Kasungu District Education Office. Informed consent was secured from all participants, either written or oral in Chichewa, through an independent facilitator for those with limited literacy. Participation was voluntary, and withdrawal was permitted at any time without penalty. In the next section, anonymity is maintained through index codes (e.g. T1 for teacher 1, P2 for parent 2).

## Results

This section presents findings from interviews and the FGD, organised around the research questions. Guided by the social model of disability and the KAP framework, the analysis demonstrates how stakeholders’ knowledge and attitudes shape inclusive education practices in a rural Malawian primary school.

### Stakeholder knowledge and inclusive education practices

This section addresses the first research question: How does stakeholder knowledge shape their inclusive education practices? The findings show that stakeholder knowledge directly influenced classroom practices, collaboration, early identification and access to support. Three distinct but related themes are presented:

- Limited pedagogical knowledge and constrained classroom practice.
- Knowledge-related role ambiguity and fragmented collaboration.
- Limited knowledge of identification and support pathways.

#### Limited pedagogical knowledge and constrained classroom practice

Across stakeholder groups, limited knowledge of inclusive education principles and pedagogical strategies constrained everyday practices. Teachers, school leaders and parents reported insufficient understanding of how to support learners with disabilities in mainstream classrooms. This knowledge gap translated into practices characterised by improvisation, uncertainty and minimal adaptation of teaching methods. Teachers described pre-service training as largely theoretical, with little emphasis on the practicalities of inclusive strategies.

As a result, classroom practices relied on trial and error instead of informed pedagogy. One teacher explained:

‘There is no clear training on how to approach these learners. We just try to manage them in class, but without a proper understanding of how to support them, it is challenging.’ (T2)

This limited pedagogical knowledge reduced teacher confidence and led to practices that prioritised classroom control over differentiated instruction. Learners with disabilities were physically present but received minimal instructional adaptation, thereby reflecting inclusion in form and not substance. The headteacher acknowledged this constraint: ‘It is unfair to expect us to manage the implementation of something we lack knowledge of’ (T1). Among parents, limited knowledge influenced enrolment decisions and engagement with schooling. Uncertainty about whether schools could adequately support children with disabilities led some parents to delay or avoid enrolment altogether: ‘We do not know if the school can handle children with disabilities properly … so we hesitate to send them’ (P4). Thus, as discussed during the FGD, limited knowledge translated into constrained instructional practices, reduced confidence and restricted access to education for learners with disabilities.

#### Knowledge-related role ambiguity and fragmented collaboration

A second way in which knowledge shaped practice was through an unclear understanding of stakeholder roles in inclusive education. School management committees, parents and community leaders perceived inclusion as the sole responsibility of teachers, thereby reflecting limited awareness of collaborative models of inclusive education. Members of school governance structures described their roles as focused on infrastructure and general school development instead of learner support: ‘We focus mostly on school development projects, but we have not been involved in discussions about how to make schools more inclusive’ (G9).

This limited knowledge of the role resulted in fragmented practices, in which inclusion-related decisions were not planned or supported collectively. Teachers, in turn, experienced isolation and role overload, as illustrated by one teacher’s comment: ‘We are expected to do everything, but we do not have clear guidelines on how to handle learners with disabilities’ (T2).

Collaboration with external professionals and between teachers was also constrained by limited knowledge of collaborative and co-teaching strategies. Teachers reported a lack of specialist support and limited opportunities for peer learning, which hindered coordinated responses to learner diversity. These findings were vindicated during the FGD as participants emphasised that limited knowledge not only affected individual practice but also weakened relational practices essential for inclusive education.

#### Limited knowledge of early identification and support pathways

A third knowledge-related constraint concerned early identification and referral practices. Stakeholders reported being able to identify only visible disabilities, with a limited understanding of learning, developmental or psychosocial difficulties. This knowledge gap resulted in delayed or absent support for many learners. One teacher explained: ‘I can only identify visible disabilities, but I am not able to know what strategies need to be applied’ (T2).

Another teacher explained: ‘For hidden disabilities, we lack the expertise to identify them … we unintentionally exclude these learners’ (T6). The absence of specialist teachers and structured referral pathways compounded this problem, thereby leaving teachers to manage complex needs without guidance. Community members were also largely excluded from identification processes because of limited awareness, as noted by the headteacher: ‘Even the community members do not participate in identifying and referring learners because they do not know how’ (T1). These findings were validated in the FGD, where a consensus across stakeholder groups was that limited knowledge at the school and community levels constrained early intervention practices. In turn, this increased the risk of marginalisation and dropout for learners with less visible disabilities.

### Stakeholder attitudes and the implementation of inclusive education

This section addresses the second research question: How do stakeholder attitudes influence inclusive education implementation? Findings show that attitudes shaped commitment, prioritisation and advocacy for inclusive education, functioning as both enablers and constraints. The following themes emerged under this question:

- Rights-based attitudes as enablers of inclusive practice.
- Conditional acceptance and attitudinal limits to inclusion.
- Cultural beliefs, stigma and their impact on practice.
- Attitudes and resource prioritisation.

#### Rights-based attitudes as enablers of inclusive practice

Many teachers and school leaders expressed strong moral and rights-based commitments to inclusive education. These attitudes motivated efforts to keep learners with disabilities in school despite severe resource constraints. One teacher stated: ‘Although it is difficult … I do not send them back home because it is their right to education’ (T1). Such attitudes translated into practices such as informal classroom adaptations, peer support arrangements and sustained enrolment of learners with disabilities. During the FGD, participants showed that supportive leadership further strengthened these practices, with the headteacher encouraging collective problem-solving and shared responsibility.

#### Conditional acceptance and attitudinal limits to inclusion

Despite general support for inclusion, attitudes shifted when learners had more severe or complex disabilities. Teachers and school leaders distinguished between mild and severe disabilities. They expressed doubt about their capacity to include learners with severe disabilities. While framed as practical concerns, these attitudes also reflected uncertainty and fear linked to limited knowledge and training, as one teacher explained: ‘We do not know how to teach them effectively in a regular classroom’ (T7). As expressed during the FGD, these conditional attitudes led to cautious or exclusionary practices, including limited instructional engagement and reliance on parents for support. This suggests that attitudes towards inclusion were shaped by perceived feasibility, leading to uneven implementation.

#### Cultural beliefs, stigma and their impact on practice

Cultural beliefs and stigma further influenced attitudes and practices. Some community members viewed disability as a curse or believed special schools were more appropriate, thereby leading to delayed enrolment or withdrawal from school. A community leader noted: ‘Some people hide the children or do not send them to school’ (C1). These attitudes affected implementation by reducing community support for inclusion and reinforcing exclusionary norms. A consensus view during the FGD was that even when learners were enrolled, stigma contributed to social isolation and limited participation, thereby undermining meaningful inclusion.

#### Attitudes and resource prioritisation

Stakeholder attitudes also influenced how resources were prioritised. Participants with strong commitments to inclusion were more likely to advocate for accessible infrastructure and learning materials. In contrast, sceptical attitudes contributed to the deprioritisation of inclusion in favour of other development needs. As one teacher explained: ‘When you do not believe in inclusion, it is hard to fight for more resources’ (T6). The FGD revealed that this feedback loop illustrates how attitudes shape practice indirectly through advocacy, planning and allocation decisions.

## Discussion

This section addresses the third research question by interpreting how stakeholder knowledge, attitudes and contextual conditions interact to shape the implementation of inclusive education in a rural Malawian primary school. Drawing on the social model of disability and the KAP model, the findings suggest that inclusive education is not constrained primarily by individual deficits but by the interaction between stakeholder agency and systemic limitations. Instead of viewing knowledge, attitudes and practices as independent factors, this study shows they are reciprocal and continually shaped by institutional and socio-economic conditions.

From a KAP perspective, stakeholder knowledge emerged as a necessary but insufficient condition for inclusive practice. Although participants generally recognised the importance of inclusive education, this knowledge rarely translated into effective classroom practice because it was constrained by limited professional development, inadequate institutional support and weak policy communication. These findings align with studies from Malawi, Ghana and Zimbabwe, which report persistent gaps between inclusive education policy and classroom implementation (De Souza 2024; Majoko 2019; Opoku et al. 2021). However, this study demonstrates that these gaps should not be understood simply as shortcomings in teacher competence. Instead, they reflect broader systemic weaknesses that limit stakeholders’ capacity to enact inclusive practices, despite their willingness. This interpretation suggests that improving knowledge alone is unlikely to produce meaningful inclusion without sustained institutional support.

The findings further indicate that stakeholder roles are negotiated in organisational structures that lack clear expectations and coordinated support. Although previous studies have reported role ambiguity in inclusive education (Ohba & Malenya 2022; Walton 2018), this case illustrates how uncertainty becomes pronounced in under-resourced rural settings where specialist services, professional collaboration and intersectoral coordination are limited. This finding shows that fragmented implementation is not simply the result of individual uncertainty but of institutional arrangements that fail to define and support collaborative responsibility. From a KAP perspective, unclear roles disrupt the relationship between knowledge and practice by weakening opportunities for collective action.

The social model of disability (Shakespeare 2010) provides further insight into why positive intentions alone did not produce inclusive practice. Participants generally expressed rights-based commitments to inclusion, but these commitments became conditional when confronted with overcrowded classrooms, inadequate resources and limited confidence in supporting learners with complex disabilities. Similar patterns have been identified in Ghana and Kenya (Ohba & Malenya 2022; Opoku et al. 2021), but the present findings suggest that these attitudes are better understood as responses to structural realities than as indicators of personal prejudice. In this context, attitudes are continually shaped by perceptions of what is practically achievable, supporting the social model’s argument that institutions and environments produce disabling conditions.

A further contribution of this study is its demonstration that attitudes influence inclusive education indirectly by shaping advocacy, decision-making and resource allocation. Stakeholders who viewed inclusion as achievable were more inclined to prioritise accessibility and learner support, whereas scepticism reduced the perceived value of investing in inclusive practices. This reciprocal relationship supports Walton’s (2018) argument that institutional support and positive beliefs support each other, creating either virtuous or vicious cycles of implementation. The findings therefore suggest that changing attitudes requires more than awareness campaigns. It also requires visible improvements in the conditions that enable successful inclusion.

Finally, the findings highlight the importance of examining inclusive education in its local context. Although weak collaboration, delayed identification and limited specialist support have been widely documented (Brydges & Mkandawire 2020; Zwane & Malale 2018), this study shows how these challenges are intensified in rural Malawi by geographic isolation, fragmented service provision and persistent socio-economic constraints. Unlike contexts in the Global North, where interprofessional collaboration is more firmly institutionalised (Ainscow 2020; Jahnukainen 2011), participants described inclusion as largely dependent on informal relationships and individual commitment. Cultural stigma and limited disability awareness further constrained community participation in identifying and supporting learners with less visible disabilities, echoing findings from Mozambique and East Africa (Franco 2023; Stone-MacDonald & Pinar-Irmak 2021). These findings validate the social model’s contention that exclusion is reproduced through systemic barriers rather than individual impairments while also demonstrating how these barriers are experienced and negotiated in a resource-constrained rural Malawian context.

### Implications and recommendations

The study contributes theoretically by demonstrating how the social model of disability and the KAP framework complement one another in explaining inclusive education as the product of interactions between stakeholder agency and structural conditions. Instead of viewing knowledge, attitudes and practices as isolated factors, the combined framework illustrates how they are continually shaped by institutional, social and material contexts. Empirically, the inclusion of multiple stakeholder perspectives provided a holistic understanding of the challenges and opportunities associated with implementing inclusive education in a rural Malawian primary school. Conducting interviews and the FGD in Chichewa further enhanced the cultural and linguistic relevance of the findings, while triangulation across data sources strengthened their trustworthiness.

The findings have important implications for policy and practice. Efforts to strengthen inclusive education in Malawi should focus on sustained implementation support. This includes practice-oriented pre-service and in-service teacher education that emphasises differentiated instruction, collaborative problem-solving and experiential learning. Clearer communication of inclusive education policies and responsibilities across teachers, school leaders, parents, community structures and government agencies is equally important to foster shared ownership of inclusion. Strengthening referral pathways through closer collaboration between the education, health and social service sectors would improve early identification and support for learners with disabilities. Equally, meaningful inclusion requires sustained investment in accessible infrastructure, assistive technologies and specialist support services. The findings also suggest that, although teacher commitment and school leadership are important enablers of inclusion, relying on individual goodwill alone cannot compensate for persistent structural and resource constraints.

Several limitations should be considered when interpreting these findings. As a qualitative single case study conducted in one rural primary school, the study was designed to generate context-specific insights instead of findings that are widely generalisable. In addition, learners (with disabilities) were not included as direct participants. Consequently, the findings reflect the perspectives of teachers, school leaders, parents, caregivers and community stakeholders and do not fully capture the lived experiences of learners themselves. Future research should therefore prioritise participatory, child-sensitive approaches that meaningfully include learners with disabilities as active contributors. Comparative studies across rural and urban settings would further enhance understanding of how inclusive education is shaped by different contextual conditions, while research designs examining learners’ educational experiences and the sustainability of community-driven inclusion initiatives would provide important evidence to inform future policy and practice.

## Conclusion

This study demonstrates that the implementation of inclusive education in a rural Malawian primary school is shaped by the dynamic interaction between stakeholder knowledge, attitudes and systemic conditions. Drawing on the social model of disability and the KAP framework, the findings show that limited professional preparation, role ambiguity, cultural beliefs and weak institutional coordination constrain stakeholders’ ability to translate inclusive policy into meaningful practice. Also, teacher commitment, supportive leadership and community engagement illustrate that inclusive practices can emerge despite severe resource limitations. The study enriches understanding of how inclusive education is negotiated in under-resourced rural contexts and highlights that exclusion is fundamentally a product of turning off social and institutional environments. Achieving meaningful inclusion therefore requires coordinated investment in teacher development, accessible infrastructure, intersectoral collaboration and context-responsive policy implementation, ensuring that inclusive education translates into equitable educational participation for all learners.

## References

[CIT0001] Abdulrahman, H.K., Adebisi, F.I., Nwako, Z. & Walton, E., 2021, ‘Revisiting (inclusive) education in the postcolony’, *Journal of the British Academy* 9(s1), 47–75. 10.5871/jba/009s1.047

[CIT0002] Ahmad, M. & Wilkins, S., 2025, ‘Purposive sampling in qualitative research: A framework for the entire journey’, *Quality & Quantity* 59(2), 1461–1479. 10.1007/s11135-024-02022-5

[CIT0003] Ainscow, M., 2020, ‘Promoting inclusion and equity in education: Lessons from international experiences’, *Nordic Journal of Studies in Educational Policy* 6(1), 7–16. 10.1080/20020317.2020.1729587

[CIT0004] Alharbi, H. & Iqtadar, S., 2024, ‘“I never feel like I am prepared enough”: Teachers’ self-efficacy, challenges and experiences teaching students with disabilities’, *Journal of Research in Special Educational Needs* 24(3), 758–770. 10.1111/1471-3802.12666

[CIT0005] Azungah, T., 2018, ‘Qualitative research: Deductive and inductive approaches to data analysis’, *Qualitative Research Journal* 18(4), 383–400. 10.1108/QRJ-D-18-00035

[CIT0006] Banda, P., 2024, ‘Exploring the knowledge and attitudes of educational stakeholders and how this influences inclusive education practices in elementary schools: A case study of a rural community in Kasungu, Malawi’, Master’s thesis, University of Cape Town, viewed 06 December 2025, from https://open.uct.ac.za/items/32c81ec3-b065-48e6-919f-70a3fbe8b231.

[CIT0007] Banks, L.M., Hunt, X., Kalua, K., Nindi, P., Zuurmond, M. & Shakespeare, T., 2022, ‘“I might be lucky and go back to school”: Factors affecting inclusion in education for children with disabilities in rural Malawi’, *African Journal of Disability* 11, a981. 10.4102/ajod.v11i0.981 PMC9724077 36483843

[CIT0008] Braun, V. & Clarke, V., 2006, ‘Using thematic analysis in psychology’, *Qualitative Research in Psychology* 3(2), 77–101. 10.1191/1478088706qp063oa

[CIT0009] Braun, V. & Clarke, V., 2023, ‘Toward good practice in thematic analysis: Avoiding common problems and becoming a knowing researcher’, *International Journal of Transgender Health* 24(1), 1–6. 10.1080/26895269.2022.2129597 PMC9879167 36713144

[CIT0010] Brydges, C. & Mkandawire, P., 2020, ‘Perceptions and experiences of inclusive education among parents of children with disabilities in Lagos, Nigeria’, *International Journal of Inclusive Education* 24(6), 645–659. 10.1080/13603116.2018.1480669

[CIT0011] Chilemba, E.M., 2013, ‘The right to primary education for children with disabilities in Malawi: A diagnosis of the conceptual approach and implementation’, *African Disability Rights Yearbook*, vol. 1, pp. 3–26, viewed 06 December 2025, from https://www.adry.up.ac.za/images/adry/volume1_2013/adry_2013_1_chapter1.pdf.

[CIT0012] Chitiyo, A. & Dzenga, C.G., 2021, ‘Special and inclusive education in Southern Africa’, *Journal of Special Education Preparation* 1(1), 55–66. 10.33043/JOSEP.1.1.55-66

[CIT0013] Cohen, L., Manion, L. & Morrison, K. (eds.), 2018, *Research methods in education*, 8th edn., Routledge, London.

[CIT0014] De Souza, B., 2022, ‘Reframing policy trajectory for inclusive education in Malawi’, *Journal of Education* 88, 68–85. 10.17159/2520-9868/i88a05

[CIT0015] De Souza, B., 2024, ‘Mitigating policy-to-practice disparities in inclusive education from a bioecological systems perspective: The case of selected secondary schools in the Lower Shire, Malawi’, *South African Journal of Education* 44(suppl. 1), S1–S10. 10.15700/saje.v44ns1a2386

[CIT0016] Engelbrecht, P., Nel, M., Smit, S. & Van Deventer, M., 2015, ‘The idealism of education policies and the realities in schools: The implementation of inclusive education in South Africa’, *International Journal of Inclusive Education* 20(5), 520–535. 10.1080/13603116.2015.1095250

[CIT0017] Florian, L., 2014, ‘What counts as evidence of inclusive education?’, *European Journal of Special Needs Education* 29(3), 286–294. 10.1080/08856257.2014.933551

[CIT0018] Franco, V., 2023, ‘School inclusion of children with disabilities in Mozambique: The teachers’ perspective’, *Frontiers in Education* 8, a1058380. 10.3389/feduc.2023.1058380

[CIT0019] Garner, P., Murray, J., Preece, D., Rose, R. & Zhao, Y., 2020, ‘Knowledge, attitudes and practices to children with disabilities in Bhutan: A contextualised literature review’, *Early Child Development and Care* 190(12), 1887–1903. 10.1080/03004430.2018.1545170

[CIT0020] Genovesi, E., Jakobsson, C., Nugent, L., Hanlon, C. & Hoekstra, R.A., 2022, ‘Stakeholder experiences, attitudes and perspectives on inclusive education for children with developmental disabilities in sub-Saharan Africa: A systematic review of qualitative studies’, *Autism* 26(7), 1606–1625. 10.1177/13623613221096208 PMC9483198 35635316

[CIT0021] Hunt, P.F., 2021, ‘Inclusive education: The case for early identification and early intervention in assistive technology’, *Assistive Technology* 33(suppl. 1), S94–S101. 10.1080/10400435.2021.1974122 34951830

[CIT0022] Jahnukainen, M., 2011, ‘Different strategies, different outcomes? The history and trends of inclusive and special education in Alberta (Canada) and in Finland’, *Scandinavian Journal of Educational Research* 55(5), 489–502. 10.1080/00313831.2010.537689

[CIT0023] Kuyini, A.B., Major, T.E., Mangope, B. & Alhassan, M., 2024, ‘Botswana teachers: Competencies perceived as important for inclusive education’, *International Journal of Inclusive Education* 28(7), 1224–1239. 10.1080/13603116.2021.1988156

[CIT0024] Lingolwe, F. & Chirwa, G.W., 2021, ‘An investigation of the influence of stakeholder factors affecting the success of the implementation of inclusive education in Malawi primary schools: A case study of four primary schools in Zomba District’, *Journal of Interdisciplinary Sciences* 5(2), 23–47.

[CIT0025] MacJessie-Mbewe, S., Ndala, K.K., Kamchedzera, E.T. & Chiwaya, P., 2023, ‘A comparative study of the implementation of Malawi national strategy on inclusive education between primary and secondary schools’, *Educational Planning* 30(2), 55–72, viewed 11 August 2025, from https://isep.info/wp-content/uploads/2023/06/230900-Journal30-2_web.pdf.

[CIT0026] Majoko, T., 2019, ‘Teacher key competencies for inclusive education: Tapping pragmatic realities of Zimbabwean special needs education teachers’, *SAGE Open* 9(1), a215824401882345. 10.1177/2158244018823455

[CIT0027] Malmqvist, J., Hellberg, K., Möllås, G., Rose, R. & Shevlin, M., 2019, ‘Conducting the pilot study: A neglected part of the research process? Methodological findings supporting the importance of piloting in qualitative research studies’, *International Journal of Qualitative Methods* 18, a1609406919878341. 10.1177/1609406919878341

[CIT0028] Mpofu, P. & Mkhatshwa, N., 2024, ‘The practicality of inclusive education in Eswatini primary schools’, *International Journal of Educational Management and Development Studies* 5(4), 158–177. 10.53378/ijemds.353123

[CIT0029] Nantongo, P.S., 2019, ‘Framing heuristics in inclusive education: The case of Uganda’s preservice teacher education programme’, *African Journal of Disability* 8, a611. 10.4102/ajod.v8i0.611 PMC6852256 31745464

[CIT0030] Ohba, A. & Malenya, F.L., 2022, ‘Addressing inclusive education for learners with disabilities in the integrated education system: The dilemma of public primary schools in Kenya’, *Compare: A Journal of Comparative and International Education* 52(1), 19–36. 10.1080/03057925.2020.1726727

[CIT0031] Opoku, M.P., Cuskelly, M., Pedersen, S.J. & Rayner, S.C., 2021, ‘Attitudes and self-efficacy as significant predictor of intention of secondary school teachers towards the implementation of inclusive education in Ghana’, *European Journal of Psychology of Education* 36, 673–691. 10.1007/s10212-020-00490-5

[CIT0032] Otukile-Mongwaketse, M., Mangope, B. & Kuyini, A.B., 2016, ‘Teachers’ understandings of curriculum adaptations for learners with learning difficulties in primary schools in Botswana: Issues and challenges of inclusive education’, *Journal of Research in Special Educational Needs* 16(3), 169–177. 10.1111/1471-3802.12069

[CIT0033] Phiri, M., 2021, ‘Challenges faced by deaf children in accessing education in Malawi’, *Deafness & Education International* 23(3), 234–249. 10.1080/14643154.2021.1952374

[CIT0034] Saloviita, T. & Consegnati, S., 2019, ‘Teacher attitudes in Italy after 40 years of inclusion’, *British Journal of Special Education* 46(4), 464–481. 10.1111/1467-8578.12286

[CIT0035] Shakespeare, T., 2010, ‘The social model of disability’, in L.J. Davis (ed.), *The disability studies reader*, pp. 214–221, Routledge, London.

[CIT0036] Slee, R., 2018, ‘Inclusive education: From policy to school implementation’, in C. Clark, A. Dyson & A. Millward (eds.), *Towards inclusive schools?* pp. 30–41, Routledge, London.

[CIT0037] Stanley, M., 2023, ‘Qualitative descriptive: A very good place to start’, in S. Nayar & M. Stanley (eds.), *Qualitative research methodologies for occupational science and occupational therapy*, pp. 52–67, Routledge, London.

[CIT0038] Stone-MacDonald, A. & Pinar-Irmak, O., 2021, ‘Inclusive education and cultural relevance in East Africa’, in N. Hamel, T. Falola, M. Berghs, A. Carothers, F. Gebrekidan, M. Nyangweso, et al. (eds.), *Disability in Africa: Inclusion, care, and the ethics of humanity*, pp. 255–285, Boydell and Brewer, Woodbridge.

[CIT0039] Tomlinson, S., 2021, ‘A sociology of special and inclusive education: Insights from the UK, US, Germany, and Finland’, in A. Köpfer, J.J.W. Powell & R. Zahnd (eds.), *International handbook of inclusive education*, pp. 59–73, Routledge, London.

[CIT0040] Tungaraza, F.D., 2014, ‘Training teachers in special needs education in Tanzania: A long and challenging ordeal to inclusion’, *Huria: Journal of the Open University of Tanzania* 16, 49–60, viewed 06 December 2025, from https://www.ajol.info/index.php/huria/article/view/108739.

[CIT0041] United Nations Educational, Scientific and Cultural Organisation (UNESCO), 2020, *Inclusion and education: All means all*, UNESCO, Paris, viewed 06 December 2025, from https://unesdoc.unesco.org/ark:/48223/pf0000373878.

[CIT0042] Walton, E., 2018, ‘Decolonising (through) inclusive education?’, *Educational Research for Social Change* 7, 31–45. 10.17159/2221-4070/2018/v7i0a3

[CIT0043] Walton, E., 2025, ‘Inclusive education: A knowledge practice based on an ecology of knowledges’, in E. Walton (ed.), *The knowledge of inclusive education: An ecological approach*, pp. 1–21, Routledge, London.

[CIT0044] Walton, E. & Engelbrecht, P., 2022, ‘Inclusive education in South Africa: Path dependencies and emergences’, *International Journal of Inclusive Education* 26, 1–19. 10.1080/13603116.2022.2061608

[CIT0045] Werning, R., Artiles, A.J., Engelbrecht, P., Hummel, M., Caballeros, M. & Rothe, A. (eds.), 2016, *Keeping the promise? Contextualising inclusive education in developing countries*, Verlag Julius Klinkhardt, Bad Heilbrunn.

[CIT0046] Zwane, S.L. & Malale, M.M., 2018, ‘Investigating barriers teachers face in the implementation of inclusive education in high schools in Gege branch, Swaziland’, *African Journal of Disability* 7(1), 1–12. 10.4102/ajod.v7i0.391 PMC6295749 30568910

